# Enlarged perivascular spaces are associated with white matter injury, cognition and inflammation in cerebral autosomal dominant arteriopathy with subcortical infarcts and leukoencephalopathy

**DOI:** 10.1093/braincomms/fcae071

**Published:** 2024-03-08

**Authors:** Nikolaos Karvelas, Bradley Oh, Earnest Wang, Yann Cobigo, Torie Tsuei, Stephen Fitzsimons, Kyan Younes, Alexander Ehrenberg, Michael D Geschwind, Daniel Schwartz, Joel H Kramer, Adam R Ferguson, Bruce L Miller, Lisa C Silbert, Howard J Rosen, Fanny M Elahi

**Affiliations:** Department of Neurology, Icahn School of Medicine at Mount Sinai, New York, NY 10029, USA; Department of Neuroscience, Icahn School of Medicine at Mount Sinai, New York, NY 10029, USA; Department of Neurology, Icahn School of Medicine at Mount Sinai, New York, NY 10029, USA; Department of Neuroscience, Icahn School of Medicine at Mount Sinai, New York, NY 10029, USA; Memory and Aging Center, Department of Neurology, UCSF Weill Institute for Neurosciences, University of California, San Francisco, CA 94158, USA; Memory and Aging Center, Department of Neurology, UCSF Weill Institute for Neurosciences, University of California, San Francisco, CA 94158, USA; Memory and Aging Center, Department of Neurology, UCSF Weill Institute for Neurosciences, University of California, San Francisco, CA 94158, USA; Department of Neurology, Icahn School of Medicine at Mount Sinai, New York, NY 10029, USA; Department of Neuroscience, Icahn School of Medicine at Mount Sinai, New York, NY 10029, USA; Memory and Aging Center, Department of Neurology, UCSF Weill Institute for Neurosciences, University of California, San Francisco, CA 94158, USA; Department of Neurology and Neurological Sciences, Stanford University, Stanford, CA 94304, USA; Memory and Aging Center, Department of Neurology, UCSF Weill Institute for Neurosciences, University of California, San Francisco, CA 94158, USA; Helen Wills Neuroscience Institute, University of California, Berkeley, Berkeley, CA 94720, USA; Innovative Genomics Institute, University of California, Berkeley, Berkeley, CA 94720, USA; Memory and Aging Center, Department of Neurology, UCSF Weill Institute for Neurosciences, University of California, San Francisco, CA 94158, USA; Advanced Imaging Research Center, Oregon Health & Science University, Portland, OR 97239, USA; Department of Neurology, Oregon Health & Science University, Portland, OR 97239, USA; Memory and Aging Center, Department of Neurology, UCSF Weill Institute for Neurosciences, University of California, San Francisco, CA 94158, USA; Department of Neurological surgery, Brain and Spinal Injury Center (BASIC), Weill Institute for Neurosciences, University of California, San Francisco, San Francisco, CA 94110, USA; San Francisco Veterans Affairs Health Care System, San Francisco, CA 94121, USA; Memory and Aging Center, Department of Neurology, UCSF Weill Institute for Neurosciences, University of California, San Francisco, CA 94158, USA; Department of Neurology, Oregon Health & Science University, Portland, OR 97239, USA; NIA-Layton Alzheimer’s Disease Research Center, Oregon Health & Science University, Portland, OR 97239, USA; Portland Veterans Affairs Health Care System, Portland, OR 97239, USA; Memory and Aging Center, Department of Neurology, UCSF Weill Institute for Neurosciences, University of California, San Francisco, CA 94158, USA; Department of Neurology, Icahn School of Medicine at Mount Sinai, New York, NY 10029, USA; Department of Neuroscience, Icahn School of Medicine at Mount Sinai, New York, NY 10029, USA; Memory and Aging Center, Department of Neurology, UCSF Weill Institute for Neurosciences, University of California, San Francisco, CA 94158, USA; James J. Peters Department of Veterans Affairs Medical Center, Bronx, NY 10468, USA

**Keywords:** CADASIL, enlarged perivascular spaces, white matter hyperintensity, cerebral small vessel disease, proteomics

## Abstract

Enlarged perivascular spaces have been previously reported in cerebral autosomal dominant arteriopathy with subcortical infarcts and leukoencephalopathy, but their significance and pathophysiology remains unclear. We investigated associations of white matter enlarged perivascular spaces with classical imaging measures, cognitive measures and plasma proteins to better understand what enlarged perivascular spaces represent in cerebral autosomal dominant arteriopathy with subcortical infarcts and leukoencephalopathy and whether radiographic measures of enlarged perivascular spaces would be of value in future therapeutic discovery studies for cerebral autosomal dominant arteriopathy with subcortical infarcts and leukoencephalopathy. Twenty-four individuals with cerebral autosomal dominant arteriopathy with subcortical infarcts and leukoencephalopathy and 24 age- and sex-matched controls were included. Disease status was determined based on the presence of *NOTCH3* mutation. Brain imaging measures of white matter hyperintensity, brain parenchymal fraction, white matter enlarged perivascular space volumes, clinical and cognitive measures as well as plasma proteomics were used in models. White matter enlarged perivascular space volumes were calculated via a novel, semiautomated pipeline, and levels of 7363 proteins were quantified in plasma using the SomaScan assay. The relationship of enlarged perivascular spaces with global burden of white matter hyperintensity, brain atrophy, functional status, neurocognitive measures and plasma proteins was modelled with linear regression models. Cerebral autosomal dominant arteriopathy with subcortical infarcts and leukoencephalopathy and control groups did not exhibit differences in mean enlarged perivascular space volumes. However, increased enlarged perivascular space volumes in cerebral autosomal dominant arteriopathy with subcortical infarcts and leukoencephalopathy were associated with increased white matter hyperintensity volume (β = 0.57, *P* = 0.05), Clinical Dementia Rating Sum-of-Boxes score (β = 0.49, *P* = 0.04) and marginally with decreased brain parenchymal fraction (β = −0.03, *P* = 0.10). In interaction term models, the interaction term between cerebral autosomal dominant arteriopathy with subcortical infarcts and leukoencephalopathy disease status and enlarged perivascular space volume was associated with increased white matter hyperintensity volume (β = 0.57, *P* = 0.02), Clinical Dementia Rating Sum-of-Boxes score (β = 0.52, *P* = 0.02), Mini-Mental State Examination score (β = −1.49, *P* = 0.03) and marginally with decreased brain parenchymal fraction (β = −0.03, *P* = 0.07). Proteins positively associated with enlarged perivascular space volumes were found to be related to leukocyte migration and inflammation, while negatively associated proteins were related to lipid metabolism. Two central hub proteins were identified in protein networks associated with enlarged perivascular space volumes: CXC motif chemokine ligand 8/interleukin-8 and C-C motif chemokine ligand 2/monocyte chemoattractant protein 1. The levels of CXC motif chemokine ligand 8/interleukin-8 were also associated with increased white matter hyperintensity volume (β = 42.86, *P* = 0.03), and levels of C-C motif chemokine ligand 2/monocyte chemoattractant protein 1 were further associated with decreased brain parenchymal fraction (β = −0.0007, *P* < 0.01) and Mini-Mental State Examination score (β = −0.02, *P* < 0.01) and increased Trail Making Test B completion time (β = 0.76, *P* < 0.01). No proteins were associated with all three studied imaging measures of pathology (brain parenchymal fraction, enlarged perivascular spaces, white matter hyperintensity). Based on associations uncovered between enlarged perivascular space volumes and cognitive functions, imaging and plasma proteins, we conclude that white matter enlarged perivascular space volumes may capture pathologies contributing to chronic brain dysfunction and degeneration in cerebral autosomal dominant arteriopathy with subcortical infarcts and leukoencephalopathy.

## Teaser

Karvelas *et al*. report that higher perivascular space (PVS) volume in cerebral autosomal dominant arteriopathy with subcortical infarcts and leukoencephalopathy (CADASIL) is associated with higher white matter hyperintensity burden, worse cognitive scores and plasma proteomic measures of inflammation. The authors conclude that imaging measures of PVS in CADASIL capture vasculo-centric inflammation.

## Graphical Abstract

**Figure:**
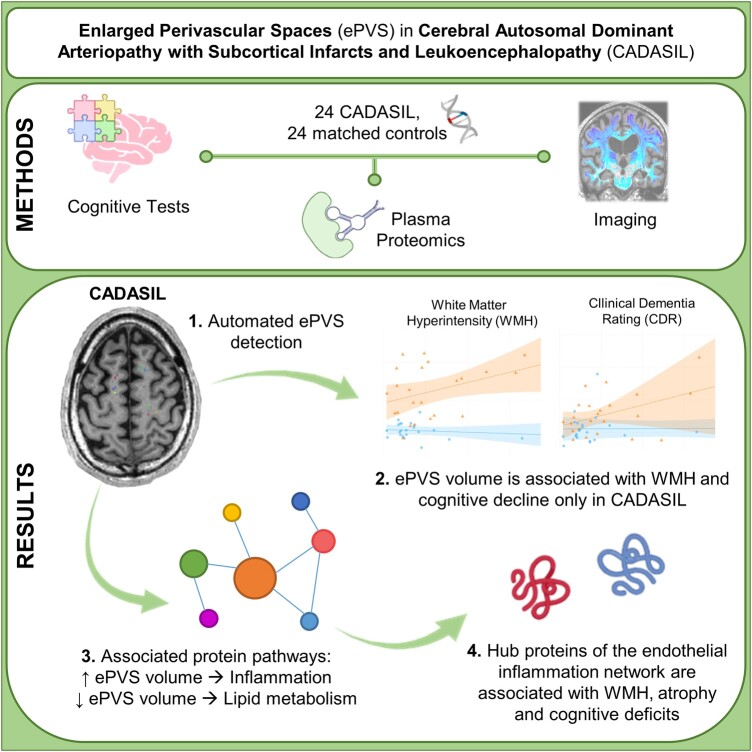

## Introduction

Considering the high prevalence of cerebral small vessel disease (cSVD), most commonly as incidental imaging findings,^1,2^ studies of vascular contributions to cognitive impairment and dementia (VCID) represent a critical area of research. A toolbox of biomarkers for accurate quantification of cSVD *in vivo* is needed for the design of impactful clinical trials and therapeutic advances.^2^ Cerebral autosomal dominant arteriopathy with subcortical infarcts and leukoencephalopathy (CADASIL), a monogenic form of VCID,^3^ provides a unique opportunity for modelling molecular and structural pathologies for the identification of biological signatures and therapeutic targets across the spectrum of VCID disease severity.

CADASIL, primarily caused by mutations in the epidermal growth factor-like repeats (EGFr) of the *NOTCH3* gene, is the most common monogenic form of vascular neurodegenerative disease, with migraines, strokes, neuropsychiatric symptoms and cognitive impairment emerging by midlife.^3^ In early stages, radiographic markers can be abnormal even in the absence of significant clinical symptoms.^4^ White matter (WM) hyperintensities (WMH) on fluid-attenuated inversion recovery (FLAIR) MRI, a classic imaging biomarker of CADASIL, are detectable years before symptom onset^4-6^; however, WMH do not consistently track with symptomatic progression of cognitive dysfunction.^7-9^ Similarly, cerebral microbleeds (CMBs), another characteristic radiographic finding, albeit less prevalent than WMH, seem to better predict stroke than cognitive decline and might be affected by metabolic factors, such as body mass index.^7,10^ Amongst radiographic biomarkers of cSVD in CADASIL, lacunes have demonstrated the most consistent association with progressive cognitive decline,^9,11,12^ although they occur in later disease stages, past the earliest desirable windows for therapeutic interventions.^3^

Perivascular spaces (PVS) are CSF-filled spaces that surround small arterioles and venules of the brain. PVS represent a major clearance route for the brain, as well as a microenvironment where immune cells and glia interact with blood vessels.^13^ Not all PVS are visible on brain imaging. The radiographically visible and quantifiable PVS are referred to as enlarged perivascular spaces (ePVS) in *in vivo* human studies.^14^ MRI-visible ePVS follow the typical course of vessels and appear isointense to CSF.^14^ It is thought that PVS enlargement can reflect structural and functional changes in cerebral microvessels and can represent the accumulation of stagnant CSF, perivascular immune cells, metabolites and proteins, including toxic substrates such as amyloid beta, in the glymphatic system along vessels.^15,16^ Pathophysiological mechanisms that are thought to influence ePVS development include increased vascular tortuosity related to dysregulated angiogenesis, changes to elasticity, blood–brain barrier (BBB) dysfunction, immune activation and changes to the extracellular matrix, as well as multifactorial clearance defects.^17^

In CADASIL, ePVS remain relatively understudied and may represent a relevant biomarker that predicts cognitive decline while capturing pathologies for future interventional trials. In this study, we use a rigorous quantitative method for measuring WM ePVS volumes to examine differences between individuals carrying *NOTCH3* mutations and age- and sex-matched controls. We focus on WM as CADASIL initially presents with WM disease and symptoms of leukoencephalopathy.^3^ We then test associations with clinically meaningful imaging and cognitive outcomes. Finally, we provide insights regarding possible implicated molecular mechanisms through associations with plasma proteomics.

## Materials and methods

### Study participants

Consecutive enrolment of CADASIL patients (*n* = 24) was undertaken from 25 February 2019 to 2 August 2021 into our prospective longitudinal study of CADASIL, VascBrain. The study was based at the UCSF Memory and Aging Center and is currently at the Icahn School of Medicine at Mount Sinai. CADASIL was confirmed based on sequencing of *NOTCH3*. Age- and sex-matched controls (*n* = 24) were sampled from ongoing longitudinal studies at the Memory and Aging Center at UCSF (Chronic Inflammation study, MarkVCID, Larry J. Hillblom foundation study, ARTFL-LEFFTDS Longitudinal Frontotemporal Lobar Degeneration Study and the MAC Alzheimer’s Disease Research Center study). All controls were selected based on normal functional status, absence of neurological disease assessed by history and physical exam or findings concerning for CADASIL. Exclusion criteria of parent studies applied to this study, including active or uncontrolled psychiatric disease such as psychosis, brain tumour or history of brain surgery. No exclusion criteria based on imaging or cognitive measures were defined, so that controls better represented the general population.

### Clinical and cognitive evaluation

A brief health history and physical examination were noted for all participants. Participants were also given a standard battery of clinical and neuropsychological tests. Clinical Dementia Rating (CDR) and CDR Sum-of-Boxes (CDR-SB) were completed on all participants via study partner interviews. For our analyses, we included CDR as a surrogate of functional impairment and measures of general cognition (Mini-Mental State Examination—MMSE), executive function (time to complete Trail Making Test B—TRAILB) and episodic visual memory (modified Rey–Osterrieth Complex Figure delayed recall at 10 min—Rey10 m).

### Neuroimaging

Participants had MRI acquired on a Siemens Tim Trio 3 Tesla scanner using the local protocol, which acquired T_1_-weighted imaging using an MP-RAGE sequence with the following parameters: 160 × 240 × 256 matrix, 160 slices, voxel size = 1 × 1 × 1 mm,^3^ flip angle 9^°^, echo time (TE) 2.98 ms and repetition time (TR) 2300 ms. The T_2_-weighted used the following parameters: 176 × 256 × 256 matrix, 176 slices, voxel size = 1 × 1 × 1 mm^3^, TE 408 ms and TR 3200 ms. The FLAIR sequence was acquired with the following parameters: 256 × 256 × 176 matrix, 176 slices, voxel size 1.0 × 1.0 × 1.0 mm^3^, TR = 5000 ms, TE 397 ms, TI 1800 ms and flip angle 120°.

Before preprocessing of the images, all T_1_-weighted images were visually inspected for quality. Images with excessive motion or other artefacts were excluded. T_1_-weighted images underwent bias field correction using the N3 algorithm, and segmentation was performed using the SPM12 (Wellcome Trust Center for Neuroimaging, London, UK) unified segmentation.^18^ A group template was generated from subject grey matter (GM) and WM tissues and CSF using the large deformation diffeomorphic metric mapping framework.^19^ Every step of the transformation was carefully inspected from the native space to the group template.

**Figure 1:**
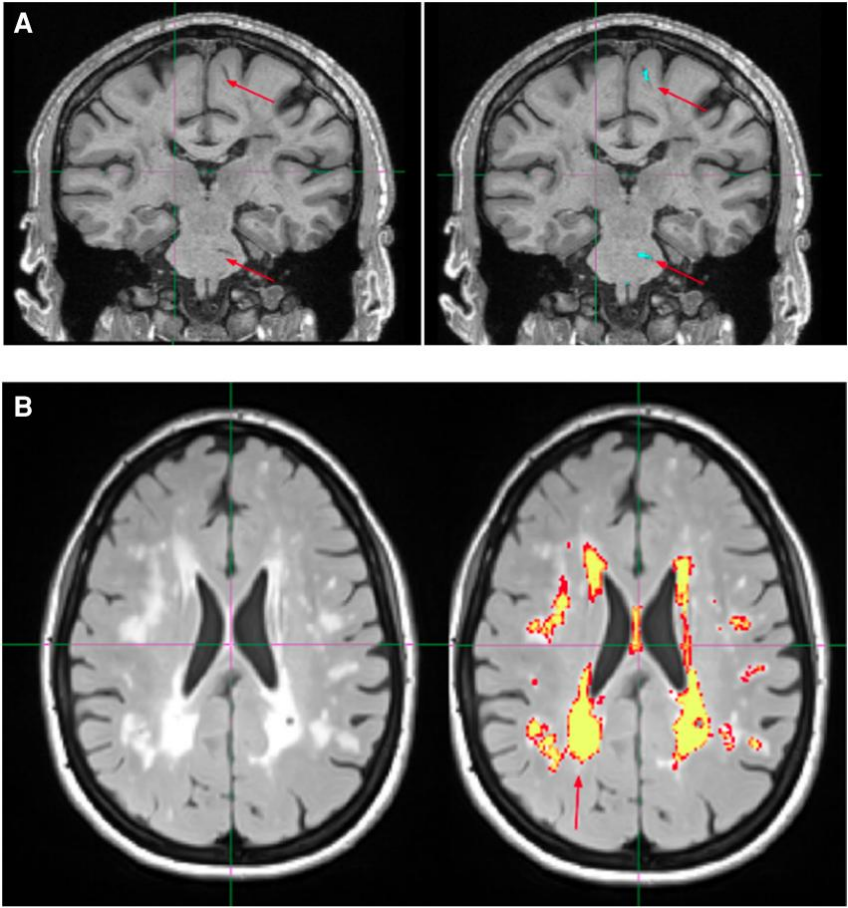
**Examples of radiological finding quantification from 2 CADASIL patients.** (**A**) Identification of WM ePVS in the coronal plane. Arrows point to visually inspected ePVS. The right image shows the result after the identification algorithm has been applied. The blue colour masking indicates autoidentified ePVS. (**B**) WMH masking in the axial plane. The yellow colour indicates high confidence of auto-identified WMH lesions, and the red colour indicates lower confidence of autoidentified WMH lesions.

An unsupervised segmentation algorithm, MRI–based multimodal autoidentification of perivascular spaces (mMAPS), previously described in the literature,^20^ was used to quantify ePVS burden within WM tissue (Fig. 1A). The STRIVE neuroimaging standards established by Wardlaw *et al*.^14^ were used in the identification and analysis of ePVS. Specifically, CSF-filled cavities in the WM with a diameter of <3 mm were categorized as ePVS. Instances with a diameter exceeding 3 mm were classified as ‘perforated ePVS’, subsequently grouped under ‘lacunes’.^14^ Total ePVS burden was quantified using a 90% binarized WM mask created in the T_1_-weighted native space, where automatic fluid-filled areas with contrast resembling CSF were identified using FSL.^21^ Corresponding T_2_-weighted images were registered into T_1_-weighted space. We enhanced the ePVS presence in the WM using the method developed in Boespflug *et al*.^20^ The proposed linear model used (i) T_1_- and T_2_-weighted images normalized by their mean values defined within the WM mask, which served as the response variable, and (ii) T_1_ and T_2_ mean values calculated in the GM and CSF, which served as nuisance and predictor variables. Correlation coefficients were calculated from CSF fluid-filled regions identified in the WM mask, such that areas of high positive value were selectively filtered as true representations of ePVS. We used an additional method, implemented in ITK, to selectively identify tortuous, vessel-like geometries from segmented, selectively filtered ePVS,^22^ by approximating the eigenvalues of the Hessian matrix at each voxel. The method factored in the modulation of eigenvalues along the principal direction of each identified ePVS to selectively filter regions of true vessel-like enlargement. An optimized Sato filter was then applied to differentiate tortuous vessel geometries of ePVS from ovoid lacunar infarcts. A density-based clustering algorithm iterated on the segmented mask to independently label unique ePVS, providing an estimate of total burden.

Visual assessment of clustered segmentation output was performed by three imaging experts (B.O., E.W., T.T.) to manually exclude false positives, including anatomical boundaries resembling tortuous geometries (e.g. sulci, cingulate, ventricles and tissue boundaries between deep GM regions) and other manifestations of small vessel disease (e.g. lacunar infarcts, ischaemic stroke infarcts and severe WM disease), as well as false negatives, including ePVS in granular subcortical areas such as the basal ganglia.

Brain parenchymal fraction (BPF) was calculated as the quotient of parenchymal tissue volume by total intracranial volume (TIV) and represents a measure of brain atrophy. WMH were segmented using FLAIR and T_1_-weighted images. We visually inspected the raw scans for quality control and visual estimation of the Fazekas scale^23^ by a board-certified neurologist (F.M.E.). In addition, the images were reviewed by a neuroradiologist to rule out other significant abnormalities. The WMH segmentation process is fully automated and based on a regression algorithm^24^ and using a hidden Markov random field with expectation maximization software^25^ (Fig. 1B). Every segmentation was visually assessed for accuracy.

### Plasma collection and proteomic analysis

Blood samples were collected in ethylenediamine tetraacetic acid (EDTA) tubes from all CADASIL patients. Samples were then centrifuged at 1000 g for 10 min, then at 2500 g for 10 min to obtain platelet-poor plasma and stored at −80°C. Afterwards, they were sent to be analysed with the SomaScan 7k assay (SomaLogic, Inc., Boulder, CO) according to standardized protocols described elsewhere.^26^ Briefly, the SomaScan assay kit employs 7596 highly selective single-stranded modified slow off-rate modified DNA aptamers (SOMAmer) for protein identification and quantification. Using a custom DNA microarray (Agilent), data are reported as relative fluorescence units (RFU). SomaLogic performed quality control, calibration and normalization of the data, and analysis of all samples was deemed appropriate. Non-human SOMAmers were removed from the data set, leaving 7363 relevant proteins for further analysis.

Due to the high dimensionality of the proteomics data, we minimized false positive associations by performing principal component analysis. We performed outlier analysis through PCA with the FactoMineR R package^27^ on standardized protein expression data that were associated with ePVS volume and removed two individuals with extreme loadings (Supplementary Fig. 1). We then reanalysed expression data with the remaining individuals. We inputted proteins of interest in the STRING database version 12.0^28^ for protein–protein functional and physical interaction analysis, the results of which were displayed as a functional network. Interactions were considered with a medium confidence score of 0.4 or higher. For further analyses, we considered only the most highly connected proteins, which were most likely to be true associations. Gene Ontology (GO) analysis of biological processes was performed through the clusterProfiler R package,^29^ which implements the Bioconductor *Homo sapiens* annotation package org.Hs.eg.db.

### Statistical analysis

Mean demographics, imaging and cognitive test measures were compared between CADASIL and control cohorts with Student’s *t*-test for continuous variables, Mann–Whitney U-test for nominal variables and Chi-square tests for categorical variables. Linear regression models were used to test for associations. For the regression models, WMH and ePVS volume were standardized using *Z*-scores in the whole sample to have a mean of 0 and a SD of 1. These models were then adjusted for age, sex and education. A different set of linear regression models was performed with the interaction between ePVS and the presence or absence of CADASIL (disease status) as the main predictor. Interaction model lines were fitted in interaction plots. Linear regression models were used to examine associations between the measures of interest and proteomic markers. Variance inflation factors (VIF) for the covariates of multivariate models were inspected to test for multicollinearity. All analyses were performed using R, version 4.2.1 (R Foundation for Statistical Computing, 2022). All statistical tests were unpaired. *P*-values for all models and correlations were corrected for false discovery rate (FDR) with the Benjamini–Hochberg method. A two-sided *P*-value ≤ 0.05 was considered statistically significant and a *P*-value < 0.10 but >0.05 marginally significant.

### Standard protocol approvals, registrations and patient consents

Study protocols were approved by the UCSF Human Research Protection Program and Institutional Review Board. Research was performed in accordance with the Code of Ethics of the World Medical Association. Written informed consent was obtained from all patients before data collection.

## Results

### Sample demographics and clinical characteristics

Participant characteristics are summarized in Table 1. The control group (*n* = 24) was selected to be age- and gender-matched to the CADASIL patient group (*n* = 24). Mean (SD) age was 50.69 (12.45) years for the CADASIL patients and 52.8 (11.26) years for the control participants. Sixteen (67%) participants in each group were female. The control group had significantly more years of educational attainment [17.09 (2.13)] than the CADASIL group [14.7 (4.17)] (*P* = 0.02).

**Table 1:** Summary of demographic, clinical and imaging characteristics

	CADASIL (*n* = 24)	Control (*n* = 24)	*P*-value
**Demographics**			
Age, (range)	50.69 ± 12.45 (23–75)	52.82 ± 11.26 (28–75)	0.54
Sex, *n* (%) female	16 (67%)	16 (67%)	1.0
Education in years	14.70 ± 4.17	17.09 ± 2.13	0.02*
**Cognitive measures**			
Clinical Dementia Rating (CDR) global score	0.17 ± 0.24	0.02 ± 0.10	0.01*
CDR Sum-of-Boxes	0.54 ± 0.83	0.15 ± 0.50	0.01*
Mini-Mental State Examination (MMSE) total score	27.72 ± 2.49	29.52 ± 0.59	0.02*
Trail B Test Time (TRAILB) (sec)	84.67 ± 65.72	47.88 ± 14.69	
Rey–Osterrieth Complex Figure Test recall at 10 min (Rey10 m)	12.24 ± 2.19	13.36 ± 2.08	0.09
**Imaging characteristics**			
Brain parenchymal fraction (BPF)	0.77 ± 0.06	0.78 ± 0.03	0.54
Total white matter hyperintensity (WMH) volume (mm^3^)	12 723.27 ± 8334.02	1306.34 ± 721	0.0000007*
Enlarged perivascular spaces (ePVS) volume (mm^3^)	547.75 ± 441.38	487.88 ± 747.55	0.73

Values are reported as mean ± standard deviation. Two-tailed Student’s *t*-tests or the non-parametric Wilcoxon rank sum was used for statistical comparison of continuous and nominal measures, and Chi-square tests were performed to compare group characteristics. *P-value < 0.05.

Concerning cognitive features, CADASIL patients had higher scores in the CDR global scale [0.17 (0.24) versus 0.02 (0.10); *P* = 0.01] and the CDR-SB score [0.54 (0.83) versus 0.15 (0.50); *P* = 0.01], had lower MMSE scores [27.72 (2.49) versus 29.52 (0.59); *P* = 0.02] and took more time to complete the TRAILB test [84.67 (65.7) versus 47.88 (14.7) seconds; *P* = 0.02]. As expected, there was a marginally significant difference for the delayed recall score of the modified Rey–Osterrieth Complex Figure test (*P* = 0.09), which was used as a surrogate of visual episodic memory.

Comparisons between imaging measures revealed no significant group differences with regard to BPF (*P* = 0.54) or ePVS volumes (*P* = 0.73). Total WMH volume was significantly increased in the CADASIL group, as expected [12 723 (8334) versus 1306 (721) mm^3^; *P* < 0.001].

### Associations of ePVS with imaging and cognitive measures

Results from all models are presented in Table 2. In the unadjusted models, increase in standardized ePVS volume was associated with increase in standardized WMH volume (β = 0.57; 95% CI = 0.01–1.14; *P* = 0.05) and in the CDRBox score (β = 0.49; 95% CI = 0.03–0.95; *P* = 0.04) for the CADASIL patient group but not controls. We also found a marginally significant decrease in BPF (β = −0.03; 95% CI = −0.06 to 0.005; *P* = 0.10) with increased ePVS volume. In the models adjusting for age, sex and education, a similar trend was observed for CDRBox (β = 0.41; 95% CI = 0.04–0.77; *P* = 0.03) but not for the other measures. Expectedly, no significant associations were found in unadjusted and adjusted models for the control group between standard ePVS volumes and any of the outcomes. Surprisingly, in the adjusted model, TRAILB completion time seemed to decrease with increased ePVS volume, but the effect was marginally significant (β = −19.72; 95% CI = −40.11 to 0.68; *P* = 0.06).

**Table 2:** Unadjusted and adjusted linear regression model associations with standardized ePVS volume as main predictor and imaging and cognitive measures as outcomes

	Unadjusted models					
	CADASIL (n = 24)		Control (n = 24)		All (n = 48)	
	β (95% CI)	*P*-value	β (95% CI)	*P*-value	β (95% CI)	*P*-value
WMH volume (scaled)	0.57 (0.01 to 1.14)	0.05*	0.006 (−0.03 to 0.04)	0.70	0.19 (−0.10 to 0.48)	0.20
BPF	−0.03 (−0.06 to 0.005)	0.10	0.001 (−0.01 to 0.01)	0.80	−0.006 (−0.02 to 0.007)	0.38
CDRBox	0.49 (0.03 to 0.95)	0.04*	−0.03 (−0.20 to 0.15)	0.77	0.12 (−0.09 to 0.32)	0.26
MMSE	−1.38 (−3.21 to 0.46)	0.13	0.11 (−0.10 to 0.32)	0.28	−0.16 (−0.76 to 0.44)	0.59
TRAILB	31.16 (−9.55 to 71.88)	0.13	−12.06 (−37.71 to 13.59)	0.33	33.36 (6.93 to 59.78)	0.01*
Rey10 m	−0.14 (−1.55 to 1.27)	0.84	−0.04 (−0.84 to 0.76)	0.91	−0.14 (−0.82 to 0.54)	0.68

	Adjusted models for age, sex and education					
WMH volume (scaled)	0.36 (−0.17 to 0.88)	0.17	0.002 (−0.03 to 0.04)	0.91	0.03 (−0.24 to 0.30)	0.80
BPF	−0.02 (−0.05 to 0.008)	0.14	0.004 (−0.006 to 0.01)	0.43	−0.003 (−0.01 to 0.009)	0.63
CDRBox	0.41 (0.04 to 0.77)	0.03*	−0.07 (−0.22 to 0.09)	0.37	0.09 (−0.09 to 0.28)	0.31
MMSE	−0.48 (−2.59 to 1.63)	0.63	0.07 (−0.17 to 0.31)	0.56	0.10 (−0.50 to 0.71)	0.73
TRAILB	20.1 (−14.66 to 54.85)	0.24	−19.72 (−40.11 to 0.68)	0.06	24.03 (−1.56 to 49.64)	0.06
Rey10 m	−0.13 (−1.73 to 1.47)	0.86	−0.008 (−0.92 to 0.90)	0.99	−0.06 (−0.81 to 0.70)	0.88

BPF, brain parenchymal fraction; CADASIL, cerebral autosomal dominant arteriopathy with subcortical infarcts and leucoencephalopathy; CDRBox, Clinical Dementia Rating Sum-of-Boxes; MMSE, Mini-Mental State Examination; Rey10 m, Rey–Osterrieth Complex Figure Test delayed recall at 10 min; TRAILB, Trail B Test completion time; WMH, white matter hyperintensity. *P-value < 0.05.

When combining both CADASIL and control participants into one group to examine disease non-specific associations, an increase in ePVS volume was associated with slowed processing speed in the unadjusted model (β = 33.36; 95% CI = 6.93–59.78; *P* = 0.01). Furthermore, the association was marginally significant in the fully adjusted model (β = 24.03; 95% CI = −1.56 to 49.64; *P* = 0.06). No other associations emerged. None of the associations were significant after FDR correction for multiple comparison. No multicollinearity was detected for any of the covariates in the multivariate models (VIF < 5).

### Associations between ePVS–CADASIL interaction with imaging and cognitive measures

**Figure 2:**
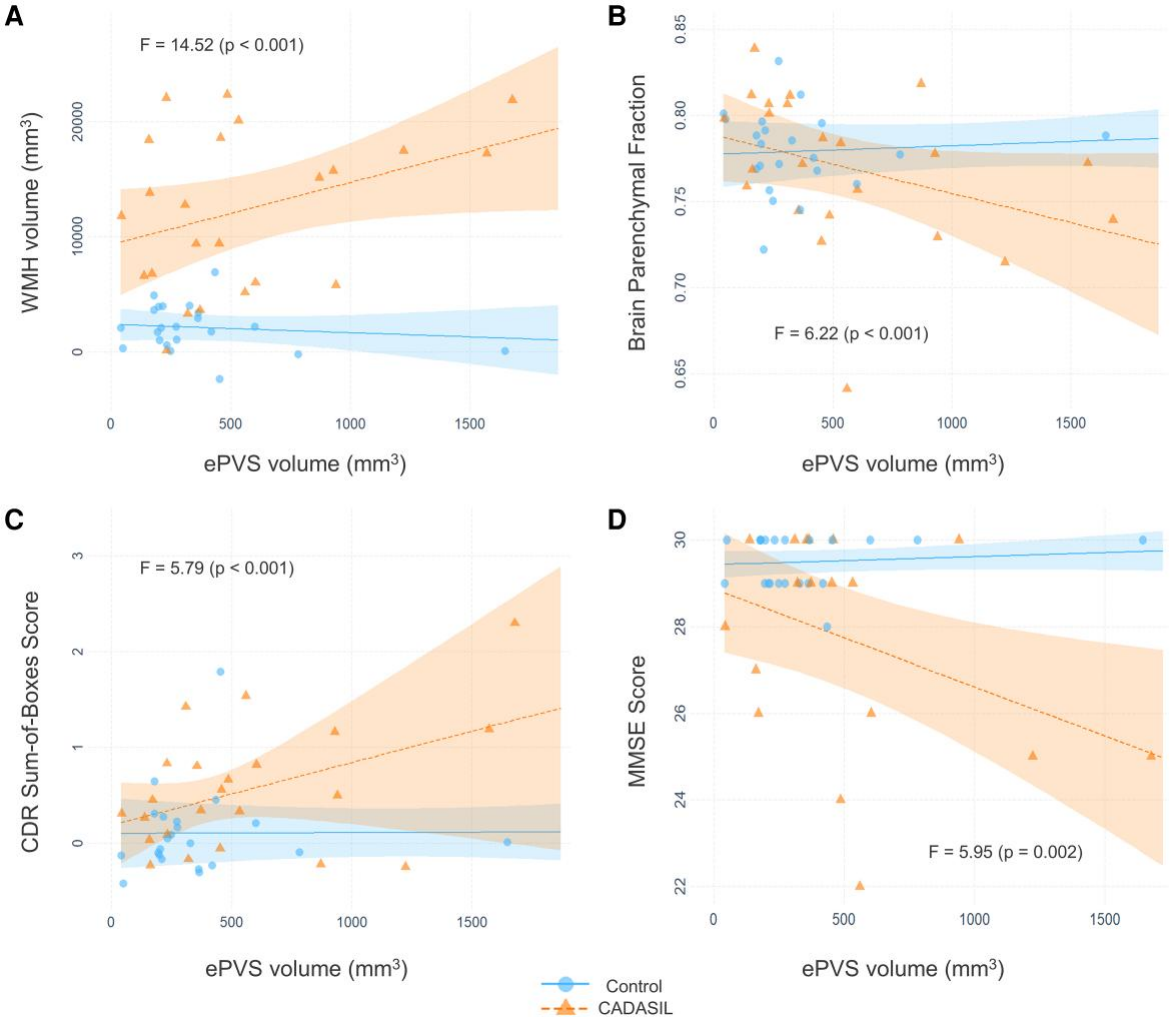
**Interaction plots with lines fitted for the CADASIL diagnosis and ePVS interaction models.** Points represent partial residuals and shaded areas 95% confidence intervals. *F*-statistics for each model are also presented. Graphs illustrate the results of (**A**) adjusted model with WMH volume as outcome, (**B**) adjusted model with BPF as outcome, (**C**) adjusted model with CDR-SB score as outcome and (**D**) unadjusted model with MMSE score as outcome. The point of one control participant with relatively large ePVS volume is not shown due to size constraints. BPF, brain parenchymal fraction; CDR, Clinical Dementia Rating; ePVS, enlarged perivascular space; MMSE, Mini-Mental State Examination; WMH, white matter hyperintensity.

Since we observed associations mainly in the CADASIL group, we proceeded to examining interaction models run in both groups combined (*n* = 48). The values derived for the interaction term between disease status and ePVS volume (CADASIL status × ePVS volume) are presented in Table 3. Some of the interaction models are visualized as interaction plots of partial residuals in Fig. 1. WMH volume was positively associated in both unadjusted (β = 0.57; 95% CI = 0.12–1.02; *P* = 0.015) and adjusted models (β = 0.45; 95% CI = 0.03–0.87; *P* = 0.03) (Fig. 2A). BPF had a marginally significant negative association with the interaction term in the unadjusted (β = −0.03; 95% CI = −0.06 to −0.002; *P* = 0.07) (Figure 2B) and in the adjusted model (β = −0.02; 95% CI = −0.05 to 0.0007; *P* = 0.06). Regarding cognitive measures, we found CDRBox and MMSE scores to be significantly associated with the interaction term. Specifically, CDRBox scores increased as interaction term values increased in the unadjusted (β = 0.52; 95% CI = 0.08–0.95; *P* = 0.02) and fully adjusted models (β = 0.39; 95% CI = 0.01–0.77; *P* = 0.04) (Fig. 2C). MMSE scores declined as interaction term values increased in the unadjusted model (β = −1.49; 95% CI = −2.81 to −0.16; *P* = 0.03) (Fig. 2D), but the effect was not statistically significant in the final model. After FDR correction, significant associations were marginally significant (all *P* = 0.06) in the unadjusted but non-significant in the adjusted models. No multicollinearity was detected for any of the covariates in the adjusted models (VIF < 5).

**Table 3:** Associations of the CADASIL diagnosis × standardized ePVS volume interaction term with imaging and cognitive measures

Outcomes	Unadjusted models		Adjusted models for age, gender and education	
	β (95% CI)	*P*-value	β (95% CI)	*P*-value
WMH volume (scaled)	0.57 (0.12 to 1.02)	0.02*	0.45 (0.03 to 0.87)	0.03*
BPF	−0.03 (−0.06 to 0.002)	0.07	−0.02 (−0.05 to 0.0007)	0.06
CDRBox	0.52 (0.08 to 0.95)	0.02*	0.39 (0.01 to 0.77)	0.04*
MMSE	−1.49 (−2.81 to −0.16)	0.03*	−1.06 (−2.43 to 0.31)	0.13
TRAILB	43.23 (−43.52 to 129.97)	0.32	9.60 (−74.41 to 96.62)	0.82
Rey10 m	−0.10 (−1.65 to 1.45)	0.90	0.06 (−1.60 to 1.72)	0.94

Unadjusted and adjusted models are presented. BPF, brain parenchymal fraction; CADASIL, cerebral autosomal dominant arteriopathy with subcortical infarcts and leucoencephalopathy; CDRBox, Clinical Dementia Rating Sum-of-Boxes; MMSE, Mini-Mental State Examination; Rey10 m, Rey–Osterrieth Complex Figure Test delayed recall at 10 min; TRAILB, Trail B Test completion time; WMH, white matter hyperintensity. *P-value < 0.05.

### Association between proteins from plasma proteomics and ePVS burden

**Figure 3:**
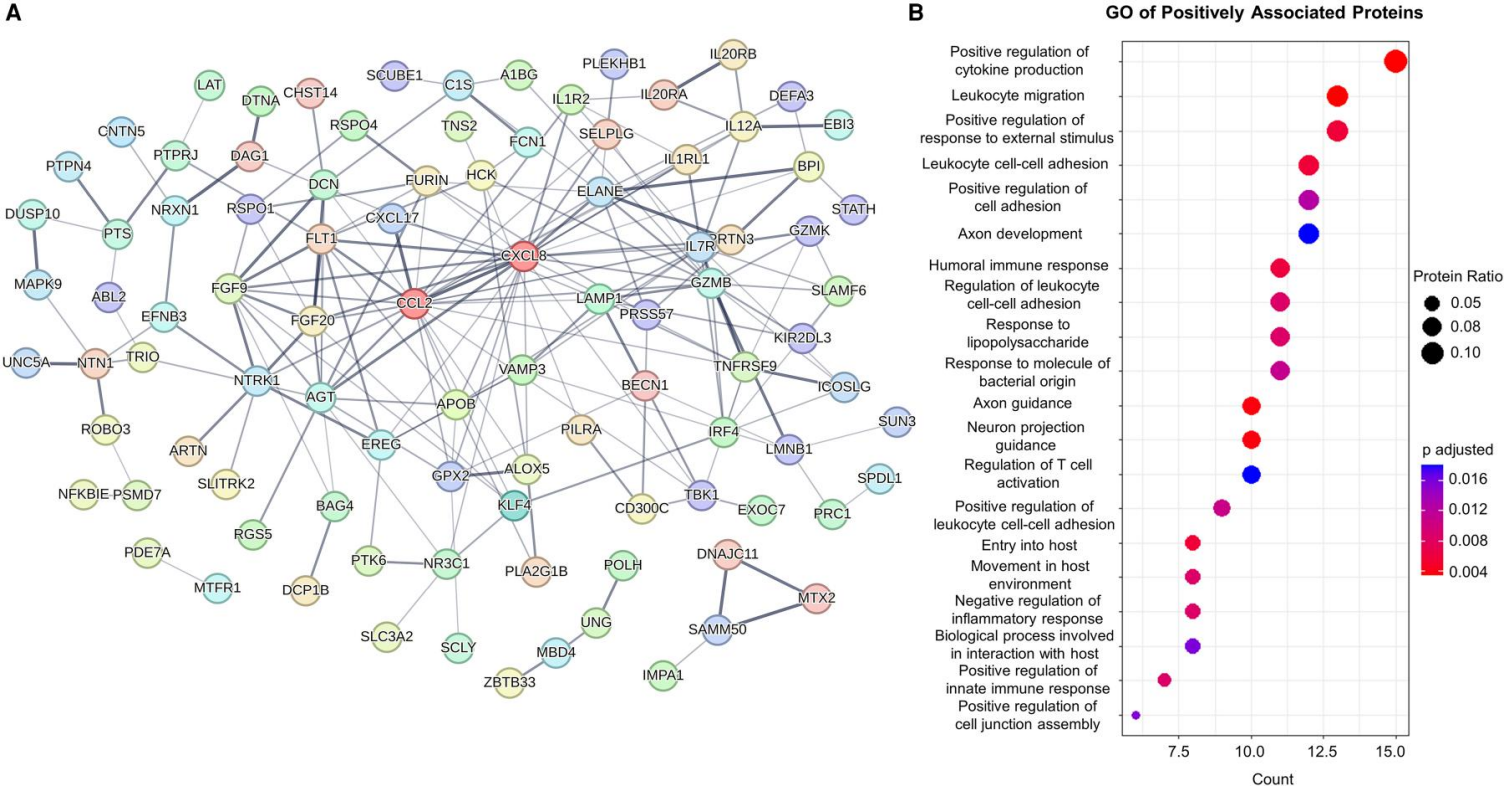
**PPI and GO analysis of proteins positively associated with ePVS volume.** (**A**) Interaction network between positively associated proteins. The proteins with most interactions (CCL2–24 edges, CXCL8–23 edges) are coloured in dark red. See Supplementary Table 1 for protein abbreviation list. (**B**) Dot plot presenting the result of BP GO analysis for the all the positively associated proteins. Intensity of edges in the interaction plots denotes confidence. Proteins with no interactions are not shown. For the GO analyses, only significant terms (adjusted *P*-value < 0.05) were included. Protein ratio is defined as the count of proteins in the term to the total number of proteins analysed. BP, biological process; GO, gene ontology.

**Figure 4:**
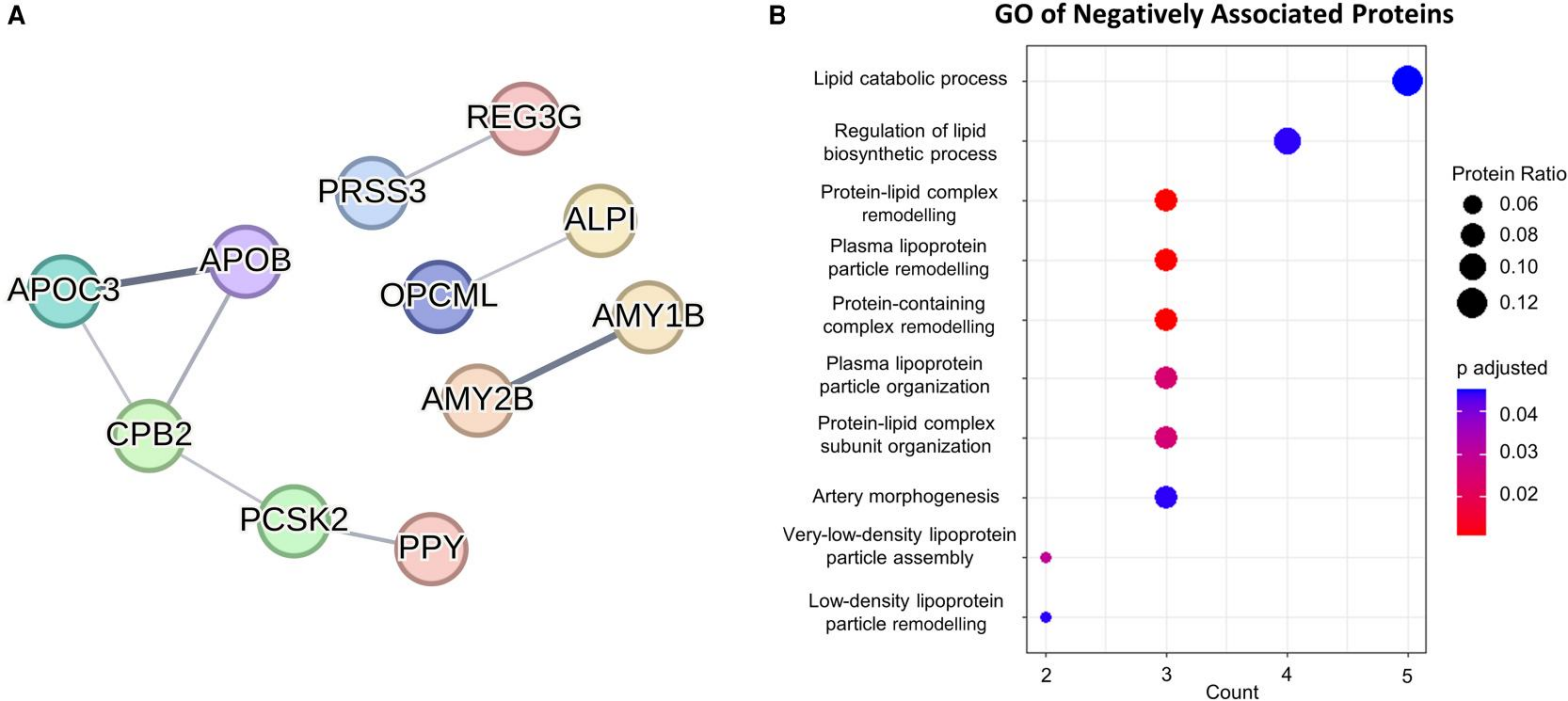
**PPI and GO analysis of proteins negatively associated with ePVS volume.** (**A**) PPI between negatively associated proteins. See Supplementary Table 2 for protein abbreviation list. (**B**) Dot plot presenting the results of BP GO analysis for the all the negatively associated proteins. Intensity of edges in the interaction plots denotes confidence. Proteins with no interactions are not shown. For the GO analyses, only significant terms (adjusted *P*-value < 0.05) were included. Protein ratio is defined as the count of proteins in the term to the total number of proteins analysed. BP, biological process; GO, gene ontology.

We hypothesized that these associations may be capturing interesting molecular mechanisms of disease in CADASIL. We proceeded to test associations of plasma protein levels, obtained through the SomaScan 7k assay, with the clinical measures of interest in the CADASIL group. A total of 194 SomaScan probes, which corresponded to 190 unique proteins, were significantly associated with ePVS volumes, out of which 145 were in the positive and 45 were in the negative direction. We then assessed protein–protein interaction networks (PPI) using the STRING database. For the positively associated proteins, 95/145 were functionally connected, and this network had a higher number of interactions than expected by chance (number of edges, 198; expected number of edges, 118; PPI enrichment *P*-value: 1.46e^−11^) (Fig. 3A). GO analysis of the biological processes revealed that the majority of enriched terms after FDR adjustment involved immune system processes, as well as neuronal axon function (Fig. 3B). We undertook a similar approach for negatively associated proteins, with 11/45 being connected significantly (number of edges, 8; expected number of edges, 4; PPI enrichment *P*-value, 0.03) (Fig. 4A). The biological process GO terms that were enriched for this subset mostly concerned lipid metabolism. Interestingly, the term for artery morphogenesis was also enriched (Fig. 4B).

### Associations between ePVS-associated proteins and clinical measures

**Figure 5:**
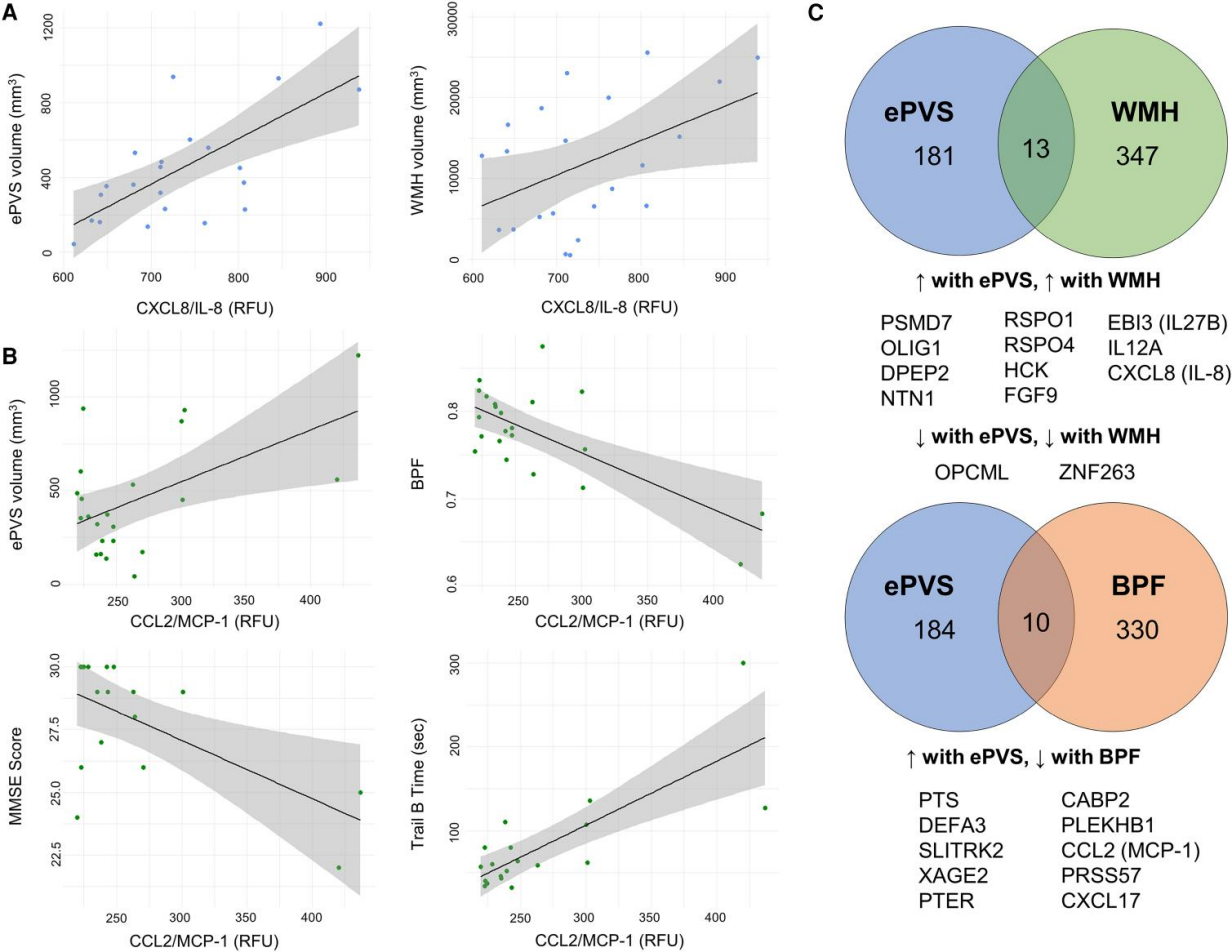
**Protein expression is associated with characteristic findings in CADASIL patients.** (**A**) Linear models with CXCL8/IL-8 as predictor and ePVS and WMH volume as outcomes. (**B**) Linear models with CCL2/MCP-1 as predictor and ePVS volume, BPF, MMSE score and TRAILB completion time as outcomes. (**C**) Overlap of proteins associated with specific neuropathological findings. Note that there was no protein associated with all three measures simultaneously. See Supplementary Table 3 for protein abbreviation list. BPF, brain parenchymal fraction; CCL2/MCP-1, C-C motif chemokine ligand 2/monocyte chemoattractant protein 1; CDRBox, Clinical Dementia Rating Sum-of-Boxes; CXCL8/IL-8, CXC motif chemokine ligand 8/interleukin-8; MMSE, Mini-Mental State Examination; Rey10 m, Rey–Osterrieth Complex Figure Test delayed recall at 10 min; RFU, relative fluorescent unit; TRAILB, Trail B Test completion time; WMH, white matter hyperintensity.

We noticed that two proteins were highly connected in our PPI networks, thereby acting as protein network hubs: C-C motif chemokine ligand 2/monocyte chemoattractant protein 1 (CCL2/MCP-1: 24 edges) and CXC motif chemokine ligand 8/interleukin-8 (CXCL8/IL-8: 23 edges) (Fig. 3A). We then examined possible associations of these hub proteins with our clinical measures of interest, presented in Fig. 5 and Table 4. Increased levels of CXCL8/IL-8 were significantly associated with increased ePVS (β = 2.44; 95% CI = 1.23–3.64; *P* = 0.0004) and WMH burden (β = 42.86; 95% CI = 3.80–81.92; *P* = 0.03) (Table 4 and Fig. 5A). An increase in CCL2/MCP-1 levels was also associated with increased ePVS levels (β = 2.77; 95% CI = 0.73 to 4.81; *P* = 0.01), TRAILB completion time (β = 0.76; 95% CI = 0.45 to 1.07; *P* = 0.00007), decreased BPF (β = −0.0007; 95% CI = −0.001 to −0.0003; *P* = 0.0003) and MMSE scores (β = −0.02; 95% CI = −0.04 to −0.007; *P* = 0.009) (Table 4 and Fig. 5B). Delayed Rey–Osterrieth Complex Figure test score was also marginally decreased (β = −0.02; 95% CI = −0.03 to 0.0006; *P* = 0.06). All associations, except WMH–CXCL8/IL-8, were significant after FDR correction.

**Table 4:** Associations of hub proteins of the ePVS-associated protein network with imaging and cognitive measures

Outcomes	CXCL8/IL-8		CCL2/MCP-1	
	β (95% CI)	*P*-value	β (95% CI)	*P*-value
ePVS (mm^3^)	2.44 (1.23 to 3.64)	0.0004*^#^	2.77 (0.73 to 4.81)	0.01*^#^
WMH (mm^3^)	42.86 (3.80 to 81.92)	0.03*	36.02 (−25.87 to 97.90)	0.24
BPF	−0.0002 (−0.0005 to 0.0001)	0.16	−0.0007 (−0.001 to −0.0003)	0.0003*^#^
CDRBox	0.0001 (−0.003 to 0.004)	0.94	0.002 (−0.001 to 0.009)	0.15
MMSE	−0.01 (−0.03 to 0.007)	0.23	−0.02 (−0.04 to −0.007)	0.009*^#^
TRAILB	0.23 (−0.12 to 0.60)	0.19	0.76 (0.45 to 1.07)	0.00007*^#^
Rey10 m	−0.001 (−0.01 to 0.01)	0.85	−0.02 (−0.03 to 0.0006)	0.06

BPF, brain parenchymal fraction; CADASIL, cerebral autosomal dominant arteriopathy with subcortical infarcts and leucoencephalopathy; CCL2/MCP-1, C-C motif chemokine ligand 2/monocyte chemoattractant protein 1; CDRBox, Clinical Dementia Rating Sum-of-Boxes; CXCL8/IL-8, CXC motif chemokine ligand 8/interleukin-8; ePVS, enlarged perivascular spaces; MMSE, Mini-Mental State Examination; Rey10 m, Rey–Osterrieth Complex Figure Test delayed recall at 10 min; TRAILB, Trail B Test completion time; WMH, white matter hyperintensity. *P-value < 0.05. ^#^Significant after Benjamini–Hochberg FDR correction.

WMH scores were not significantly associated with CCL2/MCP-1 expression (Table 4). This result prompted us to test whether there was a shared molecular marker of all three relevant patho-anatomical measures, namely ePVS, WMH and BPF. We performed linear regression models with our proteomics data as predictors and WMH and BPF as outcomes. We found significant associations with 360 and 340 SomaScan probe levels, respectively. For WMH, 13 proteins overlapped with the ePVS-associated proteins. The directionality of associations was the same for both ePVS and WMH volume (Fig. 5C). Similarly, 10 proteins were shared between the BPF- and ePVS-associated protein sets (Fig. 5C). Increased levels of all 10 proteins predicted increased ePVS volume and decreased BPF, as expected by the natural history of CADASIL. However, there was no common associated protein between all measures, suggesting degrees of biological separation between WMH volumes and brain atrophy in CADASIL. Alternatively, WMH provides a history of the brain, with signal change remaining on MRI chronically. Therefore, it is not as dynamic a measure of disease progression.

## Discussion

To date, few studies have investigated the association of MRI-visible ePVS burden with clinically meaningful outcomes in CADASIL. In this work, we used a semiautomated approach to quantify WM ePVS load in a deeply phenotyped CADASIL cohort and tested associations between ePVS volume and clinically relevant outcomes. Despite there being no significant difference in ePVS volumes between CADASIL and control participants, ePVS volume was significantly associated with increased WMH volumes, brain atrophy and declined brain function only in the CADASIL group. Additionally, we investigated plasma proteomic associations of ePVS, which were predominantly involved in inflammatory processes and lipid metabolism.

No study has examined differences in ePVS volumes between CADASIL and healthy individuals. While ePVS have been shown to increase with aging,^30^ the relationship of ePVS with clinically significant measures of brain disease remains inconclusive. Volume of ePVS *per se* may not always be an indicator of underlying disease. Higher load of ePVS can be found in healthy adolescents, mostly in the frontal and parietal WM.^31^ This phenomenon could explain the lack of a difference in mean ePVS volumes between our groups, which include participants from a wide age range. However, in the case of sporadic cSVD, which manifests most commonly in aged individuals, ePVS volumes have been shown to be increased in patients with vascular-related cognitive impairment in comparison with other types of dementia and healthy individuals,^32^ and also in patients with lacunar versus large vessel strokes.^33^ Since we only excluded patients with clinical manifestations of neurological dysfunction, some of the control participants might have harboured subclinical cSVD pathology, which could further contribute to the observed lack of difference.

In CADASIL, four studies have examined associations between clinical measures and ePVS volumes. All studies are limited by their use of visual counting methods and Likert-based categories of MRI-visible ePVS burden. The first study compared patients with few ePVS to patients with increased ePVS counts and found no differences in MMSE or modified Rankin score (mRs).^34^ In a larger study that followed a similar approach, the group of individuals with CADASIL demonstrated higher burden of ePVS and worse performance on Mattis Dementia Rating Scale (MDRS), MMSE and lower mRS than individuals with low burden of ePVS, after adjusting for age, gender and all relevant imaging markers of pathophysiology.^35^ Interestingly, in the high ePVS burden group, temporal and subinsular ePVS volumes were associated with increased WMH volume, and increased WM ePVS was marginally associated with decreased BPF.^35^ Another study found an association of ePVS in the centrum semiovale with backwards digit span (BDS), a measure of executive function, but no associations with other measures of cognition.^36^ Lastly, another group found that regional ePVS burdens in the anterior temporal lobe were associated with increased BPF, but regional ePVS were not associated with mRS score.^37^ Likert-based categorization of patients can confound results due to the variability in individual ePVS volumes. Future application of automated methods for ePVS quantification, similar to the one we implemented, can increase the reliability and comparability between studies and contribute to the standardization of measurement.

We show that WMH is associated with ePVS volume in CADASIL. WMH volume has been shown to be associated with ePVS in patients with cSVD and healthy individuals.^30,33,38^ Additionally, basal ganglia ePVS in healthy individuals have been associated with measures of increased BBB permeability.^39^ A post-mortem study in brains from CADASIL patients identified significant fibrinogen extravasation in WMH forming around ePVS, but not in WMH without other pathologies, suggesting that ePVS burden in CADASIL and the surrounding WMH may indicate BBB leakage.^40^ Moreover, from mechanistic studies, fibrinogen has been identified as a trigger of neuroinflammation and synaptic degeneration.^41^ This is reflected in the associations we found with plasma proteomic markers of immune activation, inflammation and axonal remodelling.

Our results point towards an association between ePVS volumes and brain atrophy, represented by decreased BPF, in CADASIL. Despite not achieving statistical significance, this result is indicative of a pathophysiological connection, since a similar finding has been noted in the context of various brain disorders, such as lacunar stroke^42^ and multiple sclerosis.^43^ A neuropathological study in four CADASIL patients provided experimental evidence that markedly increased ePVS burden in the basal ganglia, termed *status cribosum*, was connected with cortical neuronal apoptosis and axonal degeneration.^44^

Regarding cognitive measures, our study reinforces previous findings that increased ePVS volumes may be associated with decline in measures of functionality (CDRBox score) and general cognition (MMSE score).^35^ MMSE scores, notably, were shown to be associated with ePVS as part of the interaction term. This may be due to the larger number of patients included in the model with interaction and therefore increased power to detect this association. Furthermore, education might have confounded this model, as the control group had higher mean years of education than the CADASIL group (Table 1). The inclusion of years of education in our models attenuated the relationship of ePVS with MMSE. Although we were not powered to investigate this, cognitive reserve related to higher years of education may also be a factor to consider in the interpretation of this result. Nonetheless, a study on cSVD showed that decreased diffusion tensor image analysis along the PVS (DTI-ALPS) scores was associated with decreased MMSE scores, and DTI-ALPS mediated the relationship between WMH volume and episodic memory.^45^ The other cognitive measures studied of processing speed, executive function and visual memory were chosen based on prior observations that processing speed and executive function deteriorate early in CADASIL,^46,47^ whereas delayed memory and visuospatial abilities may exhibit decline at late stages of the disease but not consistently across patients.^46,47^ Furthermore, processing speed decline in patients with CADASIL has been associated with brain atrophy.^7^ In our study, processing speed did not associate with ePVS volumes in the CADASIL group, but we noted an association in the combined group. This is concordant with prior studies of sporadic cSVD, reporting an association of ePVS volumes with decreased processing speed and executive function scores.^48^

To connect the clinical associations of ePVS with disease pathophysiology, we investigated possible associations of ePVS volumes with plasma protein levels. Immune activation pathways, specifically leukocyte adhesion and migration, were significantly enriched in our analyses. Circulating markers of inflammation (IL-6, fibrinogen and C-reactive peptide),^49,50^ neutrophil counts and increased neutrophil-to-lymphocyte ratio^51^ have all been associated with increased ePVS burden in the general population. We identified two chemokines central to the interaction networks of ePVS-relevant proteins, CXCL8/IL-8 and CCL2/MCP-1. CXCL8/IL-8 are produced, among others, by microglia and endothelial cells,^52^ and CCL2/MCP-1, are both neutrophil and monocyte chemo-attractants.^52,53^ In individuals with normal cognition or mild cognitive impairment, these cytokines have been implicated in cognitive decline^52^ and age-related memory loss.^53^ CXCL8/IL-8 has further been associated with WMH burden^52^ and increased ischaemic stroke risk.^54^ A mechanism that has been suggested to mediate CCL2/MCP-1 effects on brain injury is the activation of microglia after acute damage and CD8^+^ T-cell recruitment,^55^ which was enriched in our GO term analysis. Interestingly, a recent article described CCL2/MCP-1 as being upregulated by aberrant Notch signalling in patients with non-alcoholic steatohepatitis, driving liver monocyte infiltration and fibrosis.^56^ The above suggests that the observed phenotypic associations in CADASIL could be of immunovascular origin; however, targeted mechanistic studies are needed to prove this.

Our study’s strengths include the age and gender matching, the deep phenotyping of individuals and the multimodal models. To our knowledge, this is the first study to connect clinical correlates of ePVS in CADASIL with molecular markers in peripheral blood. With regard to limitations, this study may have been underpowered to detect some associations, as is often the case with research of rare disorders. Studies of larger cohorts will provide additional opportunities for validating and extending our findings. In addition, cross-sectional investigations may not adequately capture ePVS abnormalities, which have been shown to be dynamic, with diurnal change.^57^ Also, we did not account for specific mutations. Patients with CADASIL mutations in EGFr 1–6 have been shown to harbour greater ePVS volume in the Anterior Temporal Lobe and subinsular regions than patients with in EGFr 7–34 mutations.^37^ Furthermore, future studies can investigate whether there is value in quantifying regional ePVS volumes in CADASIL. In the general population, regional ePVS load has been associated with specific risk factors.^30^

We found that greater ePVS volumes were associated with increased imaging measures of brain injury and degeneration, worse cognitive outcomes and markers of immune cell recruitment and inflammation in CADASIL. Our study is far from conclusive, as it mainly provides insights that should be tested in larger populations. Whether ePVS are representative on a microscopic level of inflammatory processes at the BBB, poor glymphatic flow or other aetiologies remains to be confirmed. Additional studies to further validate our reported findings and establish ePVS as a relevant marker of disease progression in CADASIL would be of great value.

## Supplementary Material

fcae071_Supplementary_Data

## Data Availability

All deidentified data employed in this study are available upon reasonable request from any qualified investigator for replication of procedures and results.

## References

[1] Das AS, Regenhardt RW, Vernooij MW, Blacker D, Charidimou A, Viswanathan A. Asymptomatic cerebral small vessel disease: Insights from population-based studies. J Stroke. 2019;21(2):121–138. 30991799 10.5853/jos.2018.03608 PMC6549070

[2] Elahi FM, Wang MM, Meschia JF. Cerebral small vessel disease-related dementia: More questions than answers. Stroke. Mar. 2023;54(3):648–660. 10.1161/STROKEAHA.122.038265 PMC10357466 36848423

[3] Chabriat H, Joutel A, Dichgans M, Tournier-Lasserve E, Bousser MG. Cadasil. Lancet Neurol. 2009;8(7):643–653. 19539236 10.1016/S1474-4422(09)70127-9

[4] Chabriat H, Levy C, Taillia H, et al Patterns of MRI lesions in CADASIL. Neurology. 1998;51(2):452–457. 9710018 10.1212/wnl.51.2.452

[5] De Guio F, Mangin JF, Duering M, Ropele S, Chabriat H, Jouvent E. White matter edema at the early stage of cerebral autosomal-dominant arteriopathy with subcortical infarcts and leukoencephalopathy. Stroke. 2015;46(1):258–261. 25370582 10.1161/STROKEAHA.114.007018

[6] Dichgans M, Filippi M, Bruning R, et al Quantitative MRI in CADASIL: Correlation with disability and cognitive performance. Neurology. 1999;52(7):1361–1367. 10227618 10.1212/wnl.52.7.1361

[7] Ling Y, De Guio F, Jouvent E, et al Clinical correlates of longitudinal MRI changes in CADASIL. J Cereb Blood Flow Metab. 2019; 39(7):1299–1305. 29400120 10.1177/0271678X18757875 PMC6668524

[8] Liem MK, van der Grond J, Haan J, et al Lacunar infarcts are the main correlate with cognitive dysfunction in CADASIL. Stroke. 2007;38(3):923–928. 17272761 10.1161/01.STR.0000257968.24015.bf

[9] Liem MK, Lesnik Oberstein SA, Haan J, et al MRI correlates of cognitive decline in CADASIL: a 7-year follow-up study. Neurology. 2009; 72(2):143–148. 19139365 10.1212/01.wnl.0000339038.65508.96

[10] Daugherty AM, Raz N. Incident risk and progression of cerebral microbleeds in healthy adults: a multi-occasion longitudinal study. Neurobiol Aging. 2017;59:22–29. 28800410 10.1016/j.neurobiolaging.2017.07.003 PMC5612885

[11] Lee JS, Choi JC, Kang SY, Kang JH, Na HR, Park JK. Effects of lacunar infarctions on cognitive impairment in patients with cerebral autosomal-dominant arteriopathy with subcortical infarcts and leukoencephalopathy. J Clin Neurol. 2011;7(4):210–214. 22259617 10.3988/jcn.2011.7.4.210 PMC3259495

[12] Jouvent E, Duchesnay E, Hadj-Selem F, et al Prediction of 3-year clinical course in CADASIL. Neurology. 2016;87(17):1787–1795. 27694265 10.1212/WNL.0000000000003252 PMC5089530

[13] Ineichen BV, Okar SV, Proulx ST, Engelhardt B, Lassmann H, Reich DS. Perivascular spaces and their role in neuroinflammation. Neuron. 2022;110(21):3566–3581. 36327898 10.1016/j.neuron.2022.10.024 PMC9905791

[14] Wardlaw JM, Smith EE, Biessels GJ, et al Neuroimaging standards for research into small vessel disease and its contribution to ageing and neurodegeneration. Lancet Neurol. 2013;12(8):822–838. 23867200 10.1016/S1474-4422(13)70124-8 PMC3714437

[15] Xue Y, Liu N, Zhang M, Ren X, Tang J, Fu J. Concomitant enlargement of perivascular spaces and decrease in glymphatic transport in an animal model of cerebral small vessel disease. Brain Res Bull. 2020;161:78–83. 32353396 10.1016/j.brainresbull.2020.04.008

[16] Perosa V, Oltmer J, Munting LP, et al Perivascular space dilation is associated with vascular amyloid-beta accumulation in the overlying cortex. Acta Neuropathol. 2022;143(3):331–348. 34928427 10.1007/s00401-021-02393-1 PMC9047512

[17] Brown R, Benveniste H, Black SE, et al Understanding the role of the perivascular space in cerebral small vessel disease. Cardiovasc Res. 2018;114(11):1462–1473. 29726891 10.1093/cvr/cvy113 PMC6455920

[18] Ashburner J, Friston KJ. Unified segmentation. Neuroimage. 2005;26(3):839–851. 15955494 10.1016/j.neuroimage.2005.02.018

[19] Ashburner J, Friston KJ. Diffeomorphic registration using geodesic shooting and Gauss-Newton optimisation. Neuroimage. 2011; 55(3):954–967. 21216294 10.1016/j.neuroimage.2010.12.049 PMC3221052

[20] Boespflug EL, Schwartz DL, Lahna D, et al MR imaging-based multimodal autoidentification of perivascular spaces (mMAPS): Automated morphologic segmentation of enlarged perivascular spaces at clinical field strength. Radiology. 2018;286(2):632–642. 28853674 10.1148/radiol.2017170205 PMC5790307

[21] Woolrich MW, Jbabdi S, Patenaude B, et al Bayesian analysis of neuroimaging data in FSL. Neuroimage. 2009;45(1 Suppl):S173–S186. 19059349 10.1016/j.neuroimage.2008.10.055

[22] Sato Y, Nakajima S, Shiraga N, et al Three-dimensional multi-scale line filter for segmentation and visualization of curvilinear structures in medical images. Med Image Anal. 1998;2(2):143–168. 10646760 10.1016/s1361-8415(98)80009-1

[23] Fazekas F, Chawluk JB, Alavi A, Hurtig HI, Zimmerman RA. MR signal abnormalities at 1.5 T in Alzheimer’s dementia and normal aging. AJR Am J Roentgenol. 1987;149(2):351–356. 3496763 10.2214/ajr.149.2.351

[24] Dadar M, Pascoal TA, Manitsirikul S, et al Validation of a regression technique for segmentation of white matter hyperintensities in Alzheimer’s disease. IEEE Trans Med Imaging. 2017;36(8):1758–1768. 28422655 10.1109/TMI.2017.2693978

[25] Avants BB, Tustison NJ, Wu J, Cook PA, Gee JC. An open source multivariate framework for n-tissue segmentation with evaluation on public data. Neuroinformatics. 2011;9(4):381–400. 21373993 10.1007/s12021-011-9109-y PMC3297199

[26] Kim CH, Tworoger SS, Stampfer MJ, et al Stability and reproducibility of proteomic profiles measured with an aptamer-based platform. Sci Rep. 2018;8(1):8382. 29849057 10.1038/s41598-018-26640-w PMC5976624

[27] Lê S, Josse J, Husson F. FactoMineR: An R package for multivariate analysis. J Stat Softw. 2008;25(1):1–18.

[28] Szklarczyk D, Kirsch R, Koutrouli M, et al The STRING database in 2023: Protein-protein association networks and functional enrichment analyses for any sequenced genome of interest. Nucleic Acids Res. Jan 6 2023;51(D1):D638–D646. 36370105 10.1093/nar/gkac1000 PMC9825434

[29] Yu G, Wang LG, Han Y, He QY. clusterProfiler: an R package for comparing biological themes among gene clusters. OMICS. 2012;16(5):284–287. 22455463 10.1089/omi.2011.0118 PMC3339379

[30] Charisis S, Rashid T, Liu H, et al Assessment of risk factors and clinical importance of enlarged perivascular spaces by whole-brain investigation in the Multi-Ethnic Study of Atherosclerosis. JAMA Netw Open. 2023;6(4):e239196. 37093602 10.1001/jamanetworkopen.2023.9196 PMC10126873

[31] Piantino J, Boespflug EL, Schwartz DL, et al Characterization of MR imaging-visible perivascular spaces in the white matter of healthy adolescents at 3 T. AJNR Am J Neuroradiol. 2020;41(11):2139–2145. 33033050 10.3174/ajnr.A6789 PMC7658833

[32] Patankar TF, Mitra D, Varma A, Snowden J, Neary D, Jackson A. Dilatation of the Virchow-Robin space is a sensitive indicator of cerebral microvascular disease: Study in elderly patients with dementia. AJNR Am J Neuroradiol. 2005;26(6):1512–1520. 15956523 PMC8149063

[33] Doubal FN, MacLullich AM, Ferguson KJ, Dennis MS, Wardlaw JM. Enlarged perivascular spaces on MRI are a feature of cerebral small vessel disease. Stroke. 2010;41(3):450–454. 20056930 10.1161/STROKEAHA.109.564914

[34] Cumurciuc R, Guichard JP, Reizine D, Gray F, Bousser MG, Chabriat H. Dilation of Virchow-Robin spaces in CADASIL. Eur J Neurol. 2006;13(2):187–190. 16490051 10.1111/j.1468-1331.2006.01113.x

[35] Yao M, Herve D, Jouvent E, et al Dilated perivascular spaces in small-vessel disease: A study in CADASIL. Cerebrovasc Dis. 2014;37(3):155–163. 24503815 10.1159/000356982

[36] Schoemaker D, Zuluaga Y, Viswanathan A, et al The INECO frontal screening for the evaluation of executive dysfunction in cerebral small vessel disease: Evidence from quantitative MRI in a CADASIL cohort from Colombia. J Int Neuropsychol Soc. 2020;26(10):1006–1018. 32487276 10.1017/S1355617720000533

[37] Hack RJ, Cerfontaine MN, Gravesteijn G, et al Effect of NOTCH3 EGFr group, sex, and cardiovascular risk factors on CADASIL clinical and neuroimaging outcomes. Stroke. 2022;53(10):3133–3144. 35862191 10.1161/STROKEAHA.122.039325 PMC9508953

[38] Rodriguez Lara F, Toro AR, Pinheiro A, et al Relation of MRI-visible perivascular spaces and other MRI markers of cerebral small vessel disease. Brain Sci. 2023; 13(9):1323. 37759924 10.3390/brainsci13091323 PMC10527297

[39] Li Y, Li M, Yang L, et al The relationship between blood-brain barrier permeability and enlarged perivascular spaces: A cross-sectional study. Clin Interv Aging. 2019;14:871–878. 31190773 10.2147/CIA.S204269 PMC6519012

[40] Rajani RM, Ratelade J, Domenga-Denier V, et al Blood brain barrier leakage is not a consistent feature of white matter lesions in CADASIL. Acta Neuropathol Commun. 2019;7(1):187. 31753008 10.1186/s40478-019-0844-x PMC6873485

[41] Merlini M, Rafalski VA, Coronado R, et al Fibrinogen induces microglia-mediated spine elimination and cognitive impairment in an Alzheimer’s disease model. Neuron. 2019;101(6):1099–1108.e6. 30737131 10.1016/j.neuron.2019.01.014 PMC6602536

[42] Zhang X, Ding L, Yang L, et al Brain atrophy correlates with severe enlarged perivascular spaces in basal ganglia among lacunar stroke patients. PLoS One. 2016;11(2):e0149593. 26900696 10.1371/journal.pone.0149593 PMC4764761

[43] Liu XY, Ma GY, Wang S, et al Perivascular space is associated with brain atrophy in patients with multiple sclerosis. Quant Imaging Med Surg. 2022;12(2):1004–1019. 35111601 10.21037/qims-21-705 PMC8739126

[44] Gray F, Polivka M, Viswanathan A, Baudrimont M, Bousser MG, Chabriat H. Apoptosis in cerebral autosomal-dominant arteriopathy with subcortical infarcts and leukoencephalopathy. J Neuropathol Exp Neurol. 2007;66(7):597–607. 17620985 10.1097/nen.0b013e318093e574

[45] Ke Z, Mo Y, Li J, et al Glymphatic dysfunction mediates the influence of white matter hyperintensities on episodic memory in cerebral small vessel disease. Brain Sci. 2022;12(12):1611. 36552071 10.3390/brainsci12121611 PMC9775074

[46] Charlton RA, Morris RG, Nitkunan A, Markus HS. The cognitive profiles of CADASIL and sporadic small vessel disease. Neurology. 2006;66(10):1523–1526. 16717212 10.1212/01.wnl.0000216270.02610.7e

[47] Buffon F, Porcher R, Hernandez K, et al Cognitive profile in CADASIL. J Neurol Neurosurg Psychiatry. 2006;77(2):175–180. 16421118 10.1136/jnnp.2005.068726 PMC2077584

[48] Passiak BS, Liu D, Kresge HA, et al Perivascular spaces contribute to cognition beyond other small vessel disease markers. Neurology. 2019;92(12):e1309–e1321. 30814324 10.1212/WNL.0000000000007124 PMC6511092

[49] Aribisala BS, Wiseman S, Morris Z, et al Circulating inflammatory markers are associated with magnetic resonance imaging-visible perivascular spaces but not directly with white matter hyperintensities. Stroke. 2014;45(2):605–607. 24399375 10.1161/STROKEAHA.113.004059 PMC3906539

[50] Ekenze O, Pinheiro A, Demissie S, et al Inflammatory biomarkers and MRI visible perivascular spaces: the Framingham Heart Study. Neurobiol Aging. 2023;127:12–22. 37018882 10.1016/j.neurobiolaging.2023.03.001 PMC10198814

[51] Jiang L, Cai X, Yao D, et al Association of inflammatory markers with cerebral small vessel disease in community-based population. J Neuroinflammation. 2022;19(1):106. 35513834 10.1186/s12974-022-02468-0 PMC9072153

[52] Gertje EC, Janelidze S, van Westen D, et al Associations between CSF markers of inflammation, white matter lesions, and cognitive decline in individuals without dementia. Neurology. 2023;100(17):e1812–e1824. 36882326 10.1212/WNL.0000000000207113 PMC10136007

[53] Bettcher BM, Neuhaus J, Wynn MJ, et al Increases in a pro-inflammatory chemokine, MCP-1, are related to decreases in memory over time. Front Aging Neurosci. 2019;11:25. 30814948 10.3389/fnagi.2019.00025 PMC6381047

[54] Georgakis MK, Gill D, Rannikmae K, et al Genetically determined levels of circulating cytokines and risk of stroke. Circulation. 2019;139(2):256–268. 30586705 10.1161/CIRCULATIONAHA.118.035905 PMC7477819

[55] Shi Z, Yu P, Lin WJ, et al Microglia drive transient insult-induced brain injury by chemotactic recruitment of CD8(+) T lymphocytes. Neuron. 2023;111(5):696–710.e9. 36603584 10.1016/j.neuron.2022.12.009

[56] Kang J, Postigo-Fernandez J, Kim K, et al Notch-mediated hepatocyte MCP-1 secretion causes liver fibrosis. JCI Insight. 2023;8(3):e165369. 36752206 10.1172/jci.insight.165369 PMC9977430

[57] Barisano G, Law M, Custer RM, Toga AW, Sepehrband F. Perivascular space imaging at ultrahigh field MR imaging. Magn Reson Imaging Clin N Am. 2021;29(1):67–75. 33237016 10.1016/j.mric.2020.09.005 PMC7694884

