# Supplementary material for: Enlarged perivascular spaces are associated with white matter injury, cognition and inflammation in cerebral autosomal dominant arteriopathy with subcortical infarcts and leukoencephalopathy
Source: Brain Commun. 2024 Mar 8;6(2):fcae071. doi: 10.1093/braincomms/fcae071 (PMC10943571; doi:10.1093/braincomms/fcae071)
Supplement: fcae071_Supplementary_Data [file fcae071_supplementary_data.pdf]

# Supplementary Material

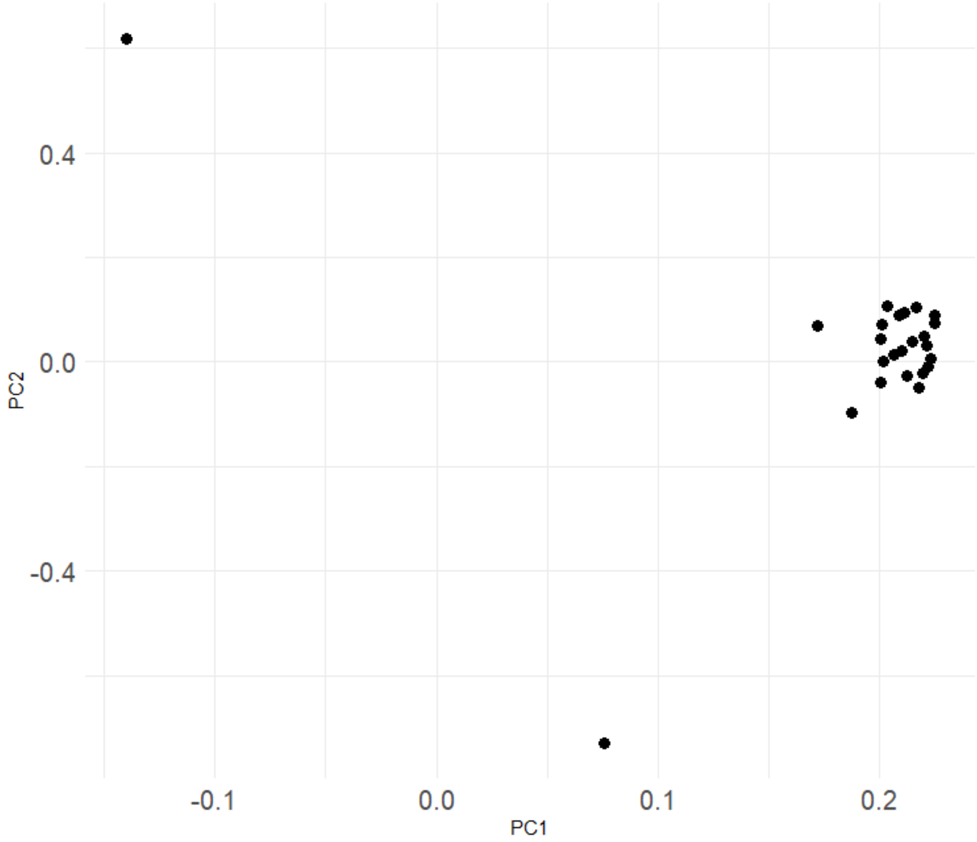


**Supplementary Figure 1.** Principal component analysis (PCA) was applied to the protein expression data of the proteins that were associated with ePVS volume, and the first two principal components (PCs) were inspected for potential outliers. Each dot represents one CADASIL patient. Two patients were separated from the rest of the cohort and were excluded from further analyses.

| Abbreviation | Full name |
|---|---|
| A1BG | alpha-1-B glycoprotein |
| ABL2 | ABL proto-oncogene 2, non-receptor tyrosine kinase |
| AGT | angiotensinogen |
| ALOX5 | arachidonate 5-lipoxygenase |
| APOB | apolipoprotein B |
| ARTN | artemin |
| BAG4 | BAG cochaperone 4 |
| BECN1 | beclin 1 |
| BPI | bactericidal permeability increasing protein |
| C1S | complement C1s |
| CCL2 | C-C motif chemokine ligand 2 |
| CD300C | CD300c molecule |
| CHST14 | carbohydrate sulfotransferase 14 |
| CNTN5 | contactin 5 |
| CXCL17 | C-X-C motif chemokine ligand 17 |
| CXCL8 | C-X-C motif chemokine ligand 8 |
| DAG1 | dystroglycan 1 |
| DCN | decorin |
| DCP1B | decapping mRNA 1B |
| DEFA3 | defensin alpha 3 |
| DNAJC11 | DnaJ heat shock protein family (Hsp40) member C11 |
| DTNA | Dystrobrevin alpha |
| DUSP10 | dual specificity phosphatase 10 |
| EBI3 | Epstein-Barr virus induced 3 |
| EFNB3 | ephrin B3 |
| ELANE | elastase, neutrophil expressed |
| EREG | epiregulin |
| EXOC7 | exocyst complex component 7 |
| FCN1 | ficolin 1 |
| FGF20 | fibroblast growth factor 20 |
| FGF9 | fibroblast growth factor 9 |
| FLT1 | fms related receptor tyrosine kinase 1 |
| FURIN | furin |
| GPX2 | glutathione peroxidase 2 |
| GZMB | granzyme B |
| GZMK | granzyme K |
| HCK | hematopoietic cell kinase |
| ICOSLG | inducible T cell costimulator ligand |
| IL12A | interleukin 12A |
| IL1R2 | interleukin 1 receptor type 2 |
| IL1RL1 | interleukin 1 receptor like 1 |
| IL20RA | interleukin 20 receptor subunit alpha |

| | |
|---|---|
| IL20RB | interleukin 20 receptor subunit beta |
| IL7R | interleukin 7 receptor |
| IMPA1 | inositol monophosphatase 1 |
| IRF4 | interferon regulatory factor 4 |
| KIR2DL3 | killer cell immunoglobulin like receptor, two Ig domains and long cytoplasmic tail 3 |
| KLF4 | KLF transcription factor 4 |
| LAMP1 | lysosomal associated membrane protein 1 |
| LAT | linker for activation of T cells |
| LMNB1 | lamin B1 |
| MAPK9 | mitogen-activated protein kinase 9 |
| MBD4 | methyl-CpG binding domain 4, DNA glycosylase |
| MTFR1 | mitochondrial fission regulator 1 |
| MTX2 | metaxin 2 |
| NFKBIE | NFKB inhibitor epsilon |
| NR3C1 | nuclear receptor subfamily 3 group C member 1 |
| NRXN1 | neurexin 1 |
| NTN1 | netrin 1 |
| NTRK1 | neurotrophic receptor tyrosine kinase 1 |
| PDE7A | phosphodiesterase 7A |
| PILRA | paired immunoglobin like type 2 receptor alpha |
| PLA2G1B | phospholipase A2 group IB |
| PLEKHB1 | pleckstrin homology domain containing B1 |
| POLH | DNA polymerase eta |
| PRC1 | protein regulator of cytokinesis 1 |
| PRSS57 | serine protease 57 |
| PRTN3 | proteinase 3 |
| PSMD7 | proteasome 26S subunit, non-ATPase 7 |
| PTK6 | protein tyrosine kinase 6 |
| PTPN4 | protein tyrosine phosphatase non-receptor type 4 |
| PTPRJ | protein tyrosine phosphatase receptor type J |
| PTS | 6-pyruvoyltetrahydropterin synthase |
| RGS5 | regulator of G protein signaling 5 |
| ROBO3 | roundabout guidance receptor 3 |
| RSPO1 | R-spondin 1 |
| RSPO4 | R-spondin 4 |
| SAMM50 | sorting and assembly machinery component 50 |
| SCLY | selenocysteine lyase |
| SCUBE1 | signal peptide, CUB domain and EGF like domain containing 1 |
| SELPLG | selectin P ligand |
| SLAMF6 | Signaling lymphocytic activation molecule family member 6 |

| | |
|---|---|
| SLC3A2 | solute carrier family 3 member 2 |
| SLITRK2 | SLIT and NTRK like family member 2 |
| SPDL1 | spindle apparatus coiled-coil protein 1 |
| STATH | statherin |
| SUN3 | Sad1 and UNC84 domain containing 3 |
| TBK1 | TANK binding kinase 1 |
| TNFRSF9 | TNF receptor superfamily member 9 |
| TNS2 | tensin 2 |
| TRIO | trio Rho guanine nucleotide exchange factor |
| UNC5A | unc-5 netrin receptor A |
| UNG | uracil DNA glycosylase |
| VAMP3 | vesicle associated membrane protein 3 |
| ZBTB33 | zinc finger and BTB domain containing 33 |

**Supplementary Table 1.** Members of the protein-protein interaction network of proteins that are positively associated with ePVS volume, presented on Figure 3. Abbreviations are in alphabetical order on the left column and full names on the right column. ePVS = enlarged Perivascular Spaces.

| **Abbreviation** | **Full name** |
|---|---|
| ALPI | alkaline phosphatase, intestinal |
| AMY1B | mylase alpha 1B |
| AMY2B | amylase alpha 2B |
| APOB | apolipoprotein B |
| APOC3 | apolipoprotein C3 |
| CPB2 | carboxypeptidase B2 |
| OPCML | opioid binding protein/cell adhesion molecule like |
| PCSK2 | proprotein convertase subtilisin/kexin type 2 |
| PPY | pancreatic polypeptide |
| PRSS3 | serine protease 3 |
| REG3G | regenerating family member 3 gamma |

**Supplementary Table 2.** Members of the protein-protein interaction network of proteins that are negatively associated with ePVS volume, presented on Figure 4. Abbreviations are in alphabetical order on the left column and full names on the right column. ePVS = enlarged Perivascular Spaces.

| Abbreviation | Full name |
|---|---|
| CABP2 | calcium binding protein 2 |
| CCL2 | C-C motif chemokine ligand 2 |
| CXCL17 | C-X-C motif chemokine ligand 17 |
| CXCL8 | C-X-C motif chemokine ligand 8 |
| DEFA3 | defensin alpha 3 |
| DPEP2 | dipeptidase 2 |
| EBI3 | Epstein-Barr virus induced 3 |
| FGF9 | fibroblast growth factor 9 |
| HCK | hematopoietic cell kinase |
| IL12A | interleukin 12A |
| NTN1 | netrin 1 |
| OLIG1 | oligodendrocyte transcription factor 1 |
| OPCML | opioid binding protein/cell adhesion molecule like |
| PLEKHB1 | pleckstrin homology domain containing B1 |
| PRSS57 | serine protease 57 |
| PSMD7 | proteasome 26S subunit, non-ATPase 7 |
| PTER | phosphotriesterase related |
| PTS | 6-pyruvoyltetrahydropterin synthase |
| SLITRK2 | SLIT and NTRK like family member 2 |
| RSPO1 | R-spondin 1 |
| RSPO4 | R-spondin 4 |
| XAGE2 | X antigen family member 2 |
| ZNF263 | zinc finger protein 263 |

**Supplementary Table 3.** Proteins commonly associated with ePVS and other imaging measures (WMH, BPF), presented on Figure 5. Abbreviations are in alphabetical order on the left column and full names on the right column. BPF = Brain Parenchymal Fraction, ePVS = enlarged Perivascular Spaces, WMH = White Matter Hyperintensities.
